# A New Cationic Porphyrin Derivative (TMPipEOPP) with Large Side Arm Substituents: A Highly Selective G-Quadruplex Optical Probe

**DOI:** 10.1371/journal.pone.0035586

**Published:** 2012-05-22

**Authors:** Li-Na Zhu, Shu-Juan Zhao, Bin Wu, Xiao-Zeng Li, De-Ming Kong

**Affiliations:** 1 Department of Chemistry, Tianjin University, Tianjin, People’s Republic of China; 2 Key Laboratory of Functional Polymer Materials, Ministry of Education, Nankai University, Tianjin, People’s Republic of China; University of Oklahoma, United States of America

## Abstract

The discovery of uncommon DNA structures and speculation about their potential functions in genes has brought attention to specific DNA structure recognition. G-quadruplexes are four-stranded nucleic acid structures formed by G-rich DNA (or RNA) sequences. G-rich sequences with a high potential to form G-quadruplexes have been found in many important genomic regions. Porphyrin derivatives with cationic side arm substituents are important G-quadruplex-binding ligands. For example, 5,10,15,20-Tetrakis(N-methylpyridinium-4-yl)-21H,23H-porphyrin (TMPyP4), interacts strongly with G-quadruplexes, but has poor selectivity for G-quadruplex versus duplex DNA. To increase the G-quadruplex recognition specificity, a new cationic porphyrin derivative, 5,10,15,20-tetra-{4-[2-(1-methyl-1- piperidinyl)ethoxy]phenyl} porphyrin (TMPipEOPP), with large side arm substituents was synthesized, and the interactions between TMPipEOPP and different DNA structures were compared. The results show that G-quadruplexes cause large changes in the UV-Vis absorption and fluorescence spectra of TMPipEOPP, but duplex and single-stranded DNAs do not, indicating that TMPipEOPP can be developed as a highly specific optical probe for discriminating G-quadruplex from duplex and single-stranded DNA. Visual discrimination is also possible. Job plot and Scatchard analysis suggest that a complicated binding interaction occurs between TMPipEOPP and G-quadruplexes. At a low [G-quadruplex]/[TMPipEOPP] ratio, one G-quadruplex binds two TMPipEOPP molecules by end-stacking and outside binding modes. At a high [G-quadruplex]/[TMPipEOPP] ratio, two G-quadruplexes bind to one TMPipEOPP molecule in a sandwich-like end-stacking mode.

## Introduction

G-quadruplexes are four-stranded nucleic acid structures formed by G-rich DNA (and RNA) sequences. In these structures, four G residues are connected by eight Hoogsteen-type hydrogen bonds to form a G-quartet plane, and several G-quartets stack to form a G-quadruplex [1]. G-rich sequences with a high potential to form G-quadruplexes are found in many important genomic regions, including telomeric repeat sequences in most eukaryotes and gene promoters in several oncogenes (for example, *c-myc, c-kit, K-ras*) [2]–[5]. The *in vivo* formation of G-quadruplex is proposed to be important within cells [5], [6], and the *in vitro* formation of G-quadruplex structures has been widely reported [7]. However, the demonstration of *in vivo* formation of G-quadruplexes remains a challenging task [8]. In fact, except for the single-stranded G-rich telomeric 3′-overhang, most G-quadruplex-forming sequences are found with their complementary strands. Depending on the conditions, these G-rich sequences can adopt different conformations, folding to G-quadruplex structures, or forming duplex structures by hybridizing with their complementary sequences. Accurate conformational detection of these gene sequences is a prerequisite for elucidating their biological functions [8]. To achieve this, a probe that specifically recognizes G-quadruplexes in the presence of duplex and single-stranded DNAs must be developed. To achieve G-quadruplex sensing *in vivo*, a specific G-quadruplex florescent probe is desirable [9].

In the past decade, efforts have been made to develop specific G-quadruplex ligands. However, few molecules show a high selectivity for G-quadruplex over duplex DNA [10], and even fewer fluorescent probes specifically recognize G-quadruplexes [9], [11]–[16]. Most reported G-quadruplex ligands bind by external π-π stacking interactions between the ligand aromatic core and the G-quartet(s) at the end(s) of the G-quadruplex. Simultaneously, the side arm substituents extending from the core interact with loops or bind the grooves of the G-quadruplex. Considering that duplex DNA has only two grooves and G-quadruplex DNA has four, a G-quadruplex ligand with a core size comparable to the G-quartet and with more than two side arms might provide good selectivity over duplex DNA [17].

Because the size of the porphyrin core is close to the G-quartet, porphyrin derivatives are important candidates in G-quadruplex ligand studies [18], [19]. Previously discovered 5,10,15,20-Tetrakis(N-methylpyridinium-4-yl)- 21H,23H-porphyrin (TMPyP4) (Scheme S1) is a well-known G-quadruplex ligand, and the binding interaction between TMPyP4 and G-quadruplexes has been extensively studied [20]–[23]. However, TMPyP4 has almost no selectivity for G-quadruplex against duplex DNA [10], [24], [25]. One reason might be that the four side arm methylpyridine substituents are too small to effectively prevent the intercalation of TMPyP4 with duplex DNA. Porphyrin derivatives with larger side arm substituents can have higher selectivity for G-quadruplex over duplex DNA, and might be more satisfactory G-quadruplex probes [26].

Based on the consideration above, we displaced the methylpyridyl substituents of TMPyP4 with larger [2-(1-methyl-1-piperidinyl) ethoxy]phenyl substituents to generate a new cationic porphyrin derivative, 5,10,15,20-tetra-{4-[2-(1-methyl-1- piperidinyl)ethoxy]phenyl} porphyrin (TMPipEOPP) (Scheme S1). This new cationic porphyrin has four bulky side arm substituents that might prevent intercalative interaction between TMPipEOPP and duplex DNA. We hypothesized that this new porphyrin would have better G-quadruplex recognizing ability than TMPyP4. UV-Vis absorption and fluorescence spectroscopic analysis showed that TMPipEOPP displayed distinctively different spectroscopic characters in the presence of G-quadruplex DNAs compared to in the presence of duplex or single-stranded DNAs, indicating that TMPipEOPP could be developed into a highly specific G-quadruplex optical probe.

## Results

### Synthesis of the new porphyrins and crystal structure of TPipEOPP•2.5MeOH

**Figure 1:**
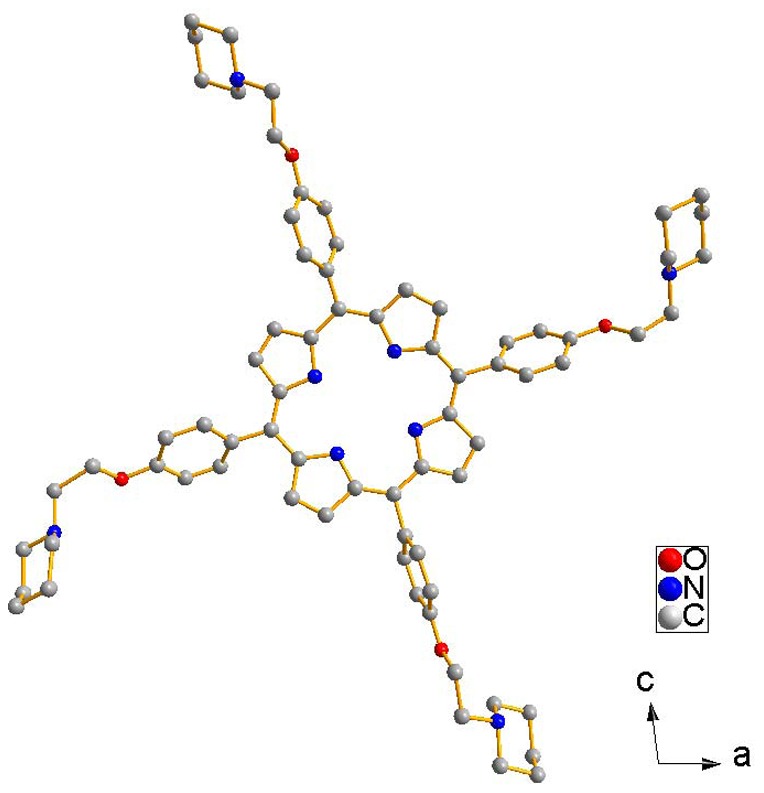
Molecular configuration for TPipEOPP•2.5MeOH. Hydrogen atoms and methanol solvent molecules are omitted for clarity.

The new cationic porphyrin TMPipEOPP were synthesized and characterized by NMR and MS. Scheme S2 shows the synthetic route. Crystal structure analysis is the best way to characterize the obtained prophyrin compound. The crystal structure of TMPipEOPP was not obtained till now, but purple-brown crystals of its precursor product, TPipEOPP•2.5MeOH, were obtained, and the crystal structure was characterized. This can provide important information for the characterization of TMPipEOPP. The skeletal framework of the TPipEOPP molecule is shown in Figure 1. The molecule is asymmetrical. The four pyrrole nitrogen atoms are approximately co-planar with an average core size (Ct•••N) of 2.08 Å. The porphyrin macrocycle has a ruffled shape. The four pyrrole rings alternate up and down out of the above plane with dihedral angles of 7.298–12.469°. The four *meso* side arms are suspended from the core porphyrin macrocycle. The twist angles between the four benzene rings and the porphyrin plane are around 60°. The adjacent side arms twist against the porphyrin plane in the opposite direction. The side arms are flexible with respect to the ethyl group. All the piperidine rings adopt a chair conformation. The dihedral angles between the cover of the chair and the corresponding phenoxyl group are 74.703, 84.922, 61.976, 75.082°. Crystal data and selected bond lengths and angles are in Table S1.

### Effects of DNAs on the TMPipEOPP UV-Vis absorption spectrum

The aim of this work was to develop a specific G-quadruplex probe, so the interaction between the cationic porphyrin TMPipEOPP and DNA was studied. We investigated the interactions between TMPipEOPP and ten different DNAs (Table 1). These ten DNAs could be divided into three groups. Group I was Hum24, KRAS, M3Q and Oxy28, four G-rich oligonucleotides whose sequences are from the human or animal genomes. Hum24 and Oxy28 have repeated subunits of human and Oxytricha telomeres. KRAS is a 32-nucleotide sequence in the promoter of the human KRAS gene. M3Q is a 20-nucleotide sequence upstream of the mRNA initiation codon of MT3-MMP. The formation of G-quadruplex structures by these four oligonculeotides was confirmed by circular dichroism (CD) spectroscopy (Figure S1). The results of CD spectroscopy indicated that KRAS and M3Q formed parallel G-quadruplexes, Oxy28 formed an antiparallel G-quadruplex, and Hum24 formed a mixed parallel/antiparallel G-quadruplex. Group II also included four DNAs. AT, GC and LD formed short-stranded duplex structures. Calf thymus DNA (CtDNA) is a well-known long-stranded duplex DNA. Group III included two oligonucleotides: ssDNA1 and ssDNA2, both of which existed in single-stranded form.

**Table 1:** The oligonucleotides used in this work.

Entry	Abbreviation	Sequence	Extinction coefficient [L·mol^−1^·cm^−1^]	Structure
1	Hum24	5′-(TTAGGG)_4_	244600	G-quadruplex
2	M3Q	5′- GA(GGGA)_3_GAGGGA	222500	G-quadruplex
3	KRAS	5′-AGGGCGGTGTGGGAAGAGGGAAGAGGGGGAGG	341000	G-quadruplex
4	Oxy28	5′-(GGGGTTTT)_3_GGGG	262000	G-quadruplex
5	AT	5′-(AT)_6_	133300	Duplex
6	GC	5′-(GC)_6_	101100	Duplex
7	LD	5′-GCGCAATTGCGC	108700	Duplex
8	CtDNA			Duplex
9	ssDNA1	5′-GAGCTCTCGAAAGAGCT CCGATTA	235800	Single-stranded
10	ssDNA2	5′-TAGAGCACACCTGTCCG TG	189400	Single-stranded

**Figure 2:**
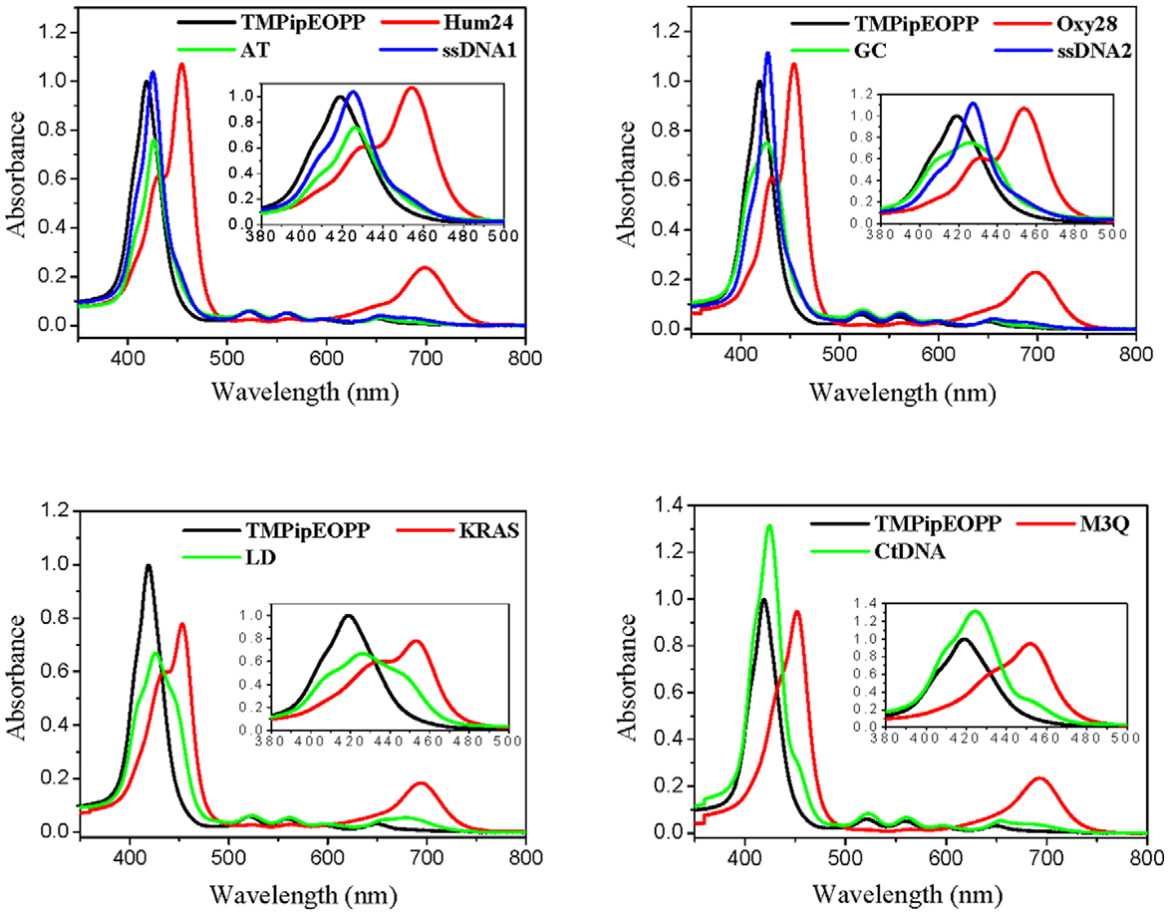
UV-Vis absorption spectra of TMPipEOPP in the absence or presence of different DNAs. [TMPipEOPP] = 5 µM. [DNA] = 10 µM (strand concentration). [CtDNA] = 240 µM (base concentration). The inserts show the absorption spectra in the range of 380–500 nm.

UV-Vis spectroscopy is an important tool in the studies of small molecule-DNA interactions. As shown in Figure 2, the absorption spectrum of free TMPipEOPP has a strong Soret band centered at 419 nm and four weak absorption bands centered at 520, 559, 593 and 649 nm, respectively. In the presence of 10 µM DNA, the absorption spectrum of TMPipEOPP changed, depending on the DNA structure. Addition of G-quadruplexes (Hum24, KRAS, M3Q and Oxy28) caused an obvious hypochromicity and red shift (∼11 nm) at the Soret band (419 nm) of TMPipEOPP, accompanied by the appearance of a strong new absorption band centered around 455 nm. Simultaneously, notable hypochromicities were observed for the weak peaks at 520 and 559 nm, and a new band with much stronger absorption intensity appeared around 700 nm, with the weak bands at 593 and 649 nm caged in this new band.

The effects of duplex DNAs (AT, GC, LD and CtDNA) on the absorption bands of TMPipEOPP were much weaker than the G-quadruplex DNAs. These DNAs caused only a slight red shift (about 7 nm) for the TMPipEOPP Soret band, with no new absorption bands around 455 nm; only a shoulder peak appeared around 450 nm in the presence of LD and CtDNA. Although a new band centered around 682 nm appeared with LD, its absorption intensity was much weaker than bands caused by G-quadruplexes.

The addition of single-stranded DNAs (ssDNA1 and ssDNA2) caused red shifts of the TMPipEOPP Soret band from 419 to 426 nm, but was accompanied by increases in absorption intensities. No new bands were observed, and the bands at 520, 559, 593 and 649 nm were almost unaffected.

Overall, compared with duplex and single-stranded DNAs, G-quadruplexes caused characteristic changes in the TMPipEOPP absorption spectrum: (1) Addition of G-quadruplexes casued an obvious hypochromicity and red shift at the Soret band of TMPipEOPP, and a new band appeared around 455 nm. The new band became the strongest band at last. However, in the presence of duplex or single-stranded DNAs, the previous Soret band was still the strongest band. (2) The absorption bands at 520 and 559 nm greatly decreased compared to free TMPipEOPP. However, the presence of duplex or single-stranded DNAs had no effect on these two bands. (3) A new absorption band appeared at around 700 nm, and the new band became the second strongest absorption band. No such a new band appeared in the presence of duplex or single-stranded DNAs. Taking all factors into consideration, TMPipEOPP could be used as a good colorimetric probe to discriminate G-quadruplex from duplex and single-stranded DNA. TMPyP4 is reported to bind strongly to the minor groove of AT-rich duplex DNAs [27], but the presence of the duplex DNA AT had almost no much effect on the TMPipEOPP absorption spectrum, indicating that the introduction of bulky cationic side arms surrounding the aromatic core of TMPipEOPP hampered the interaction of TMPipEOPP with duplex DNA. TMPipEOPP also discriminated between G-quadruplex and long-stranded duplex DNA (CtDNA). This is important for G-quadruplex probes, but few G-quadruplex probes give good results [28].

**Figure 3:**
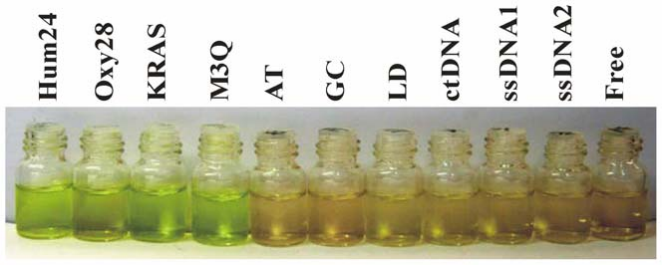
G-quadruplex discrimination from duplex and single-stranded DNA by the naked eyes. The DNA used in each tube is labeled at the top of the figure. [TMPipEOPP] = 5 µM. [DNA] = 10 µM (strand concentration). [CtDNA] = 240 µM (base concentration).

Since G-quadruplexes cause greater absorption spectrum changes in TMPipEOPP than duplex and single-stranded DNAs, we wondered if TMPipEOPP could be used as a colorimetric probe for visual discrimination of G-quadruplexes from duplex and single-stranded DNAs. As shown in Figure 3, a solution of free TMPipEOPP was reddish orange. With the addition of duplex and single-stranded DNAs, the solution color was nearly unchanged. However, solutions containing G-quadruplexes gave a distinct green-yellow color. These results indicated that TMPipEOPP could be used as a G-quadruplex probe for the visual recognition of G-quadruplexes.

### Effects of DNAs on the TMPipEOPP fluorescence spectrum

**Figure 4:**
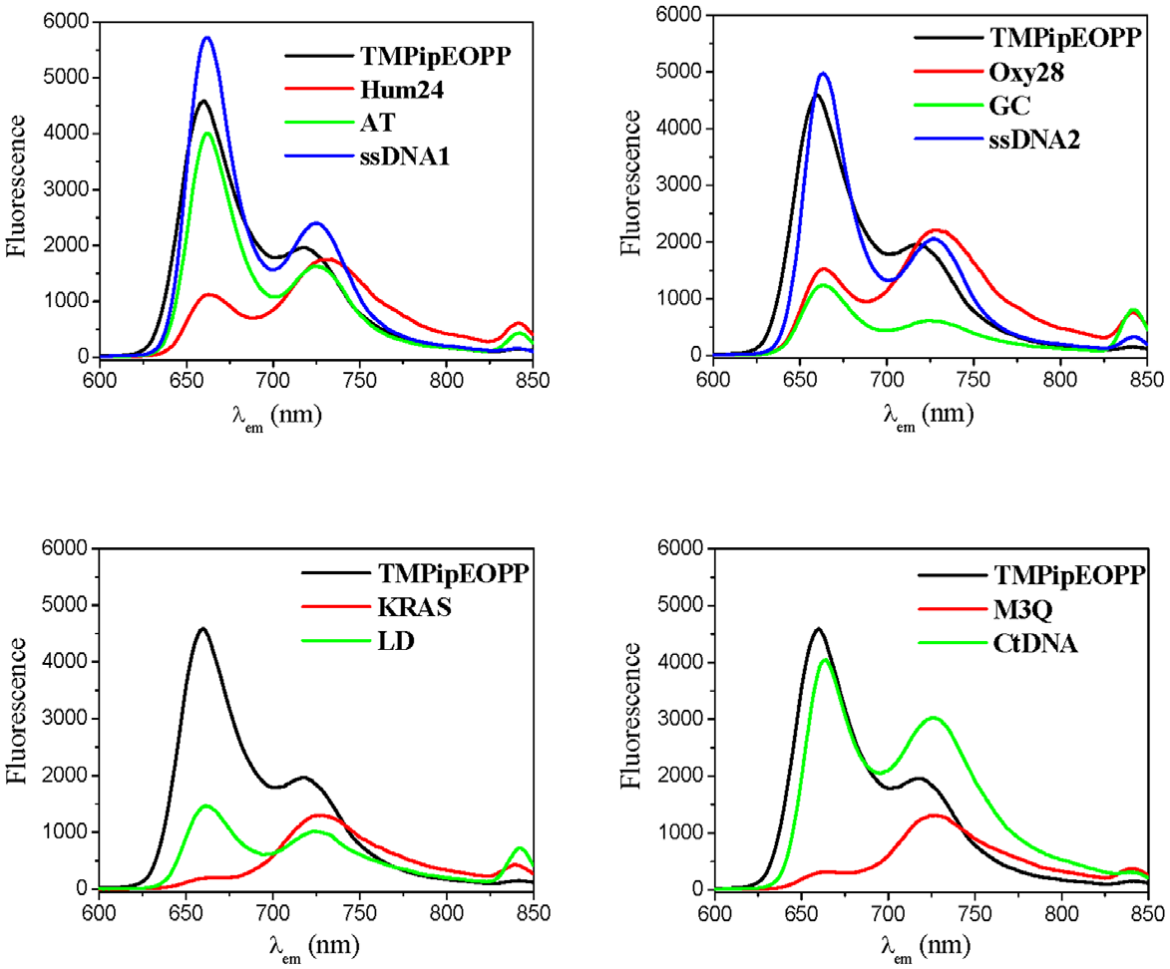
Fluorescence spectra of TMPipEOPP in the absence or presence of different DNAs when excited at 422 nm. [TMPipEOPP] = 5 µM. [DNA] = 10 µM (strand concentration). [CtDNA] = 240 µM (base concentration).

The effects of the ten DNAs on TMPipEOPP fluorescence were also compared. At the excitation wavelength of 422 nm, the fluorescence spectrum of TMPipEOPP displayed two peaks at 660 nm and 726 nm (Figure 4), with the fluorescence intensity of the 660-nm peak much stronger than the 726-nm peak. The addition of 10 µM duplex or single-stranded DNA had little effect on the shape of the emission spectrum: the fluorescence intensity at the lower wavelength (661–663 nm) was still much higher than the higher wavelength (725–730 nm). However, the opposite result was observed in the presence of G-quadruplexes. The fluorescence intensity at the higher wavelength was higher than the intensity at the lower wavelength. In the presence of M3Q and KRAS, the emission peak at the lower wavelength nearly disappeared. These observations suggested that the main emission peak might move from around 660 nm to 726 nm in the presence of G-quadruplexes.

**Figure 5:**
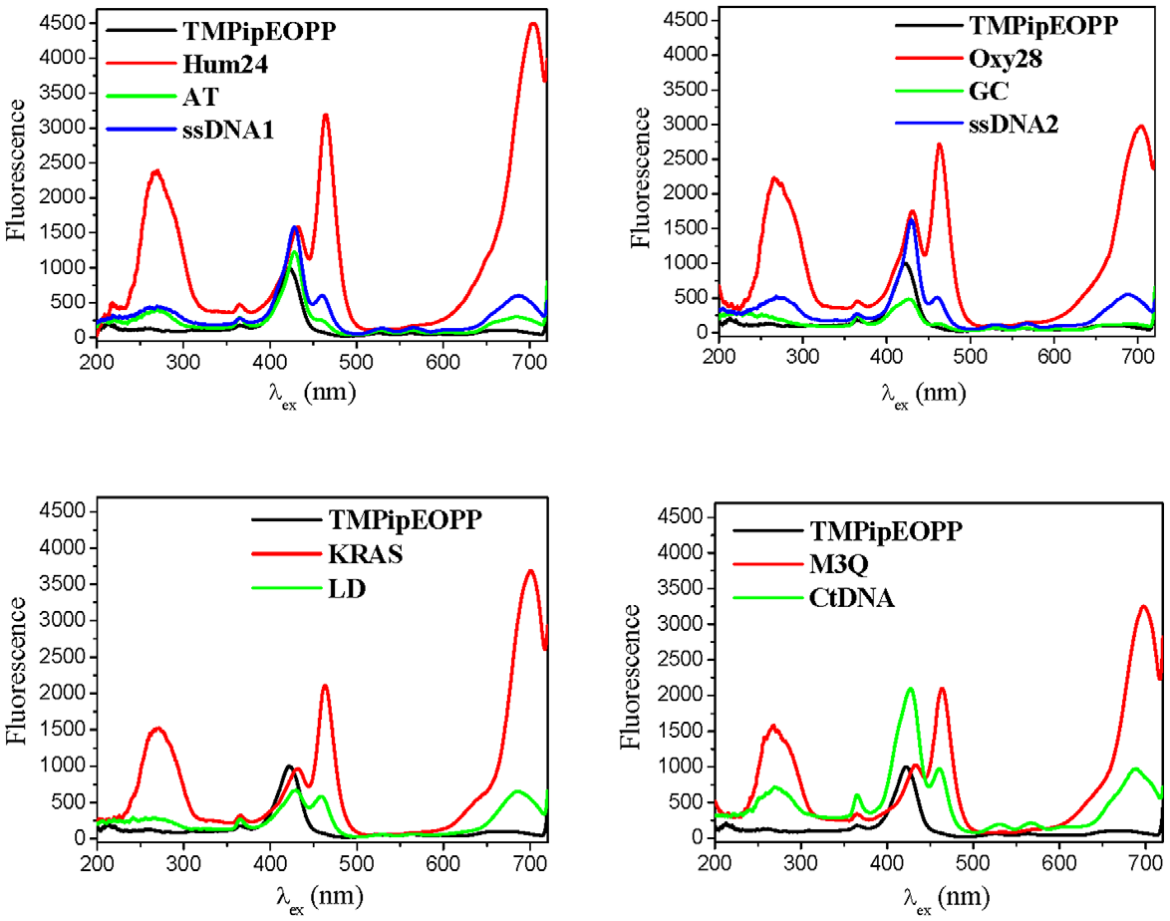
Excitation spectra of TMPipEOPP in the absence or presence of different DNAs when the emission wavelength is held at 726 nm. [TMPipEOPP] = 5 µM. [DNA] = 10 µM (strand concentration). [CtDNA] = 240 µM (base concentration).

Then, the effects of the ten DNAs on the TMPipEOPP excitation spectrum were investigated, keeping the emission wavelength at 726 nm. Free TMPipEOPP showed a single excitation peak at 422 nm (Figure 5). However, in the presence of the four G-quadruplexes, three strong new excitation peaks appeared at around 270, 464 and 700 nm, respectively. Although the previous excitation peak of TMPipEOPP was also observed, a red shift from 422 nm to around 432 nm was also observed, with an intensity much lower than the peak at 464 nm. In the presence of duplex or single-stranded DNAs, the previous excitation peak of TMPipEOPP (red shift to around 426) was still the strongest excitation peak. These results indicated that the presence of G-quadruplexes led to the appearance of three new excitation peaks, and these three peaks (around 270, 464 and 700 nm), rather than the previous excitation peak (422 nm), became the main excitation peaks of TMPipEOPP.

**Figure 6:**
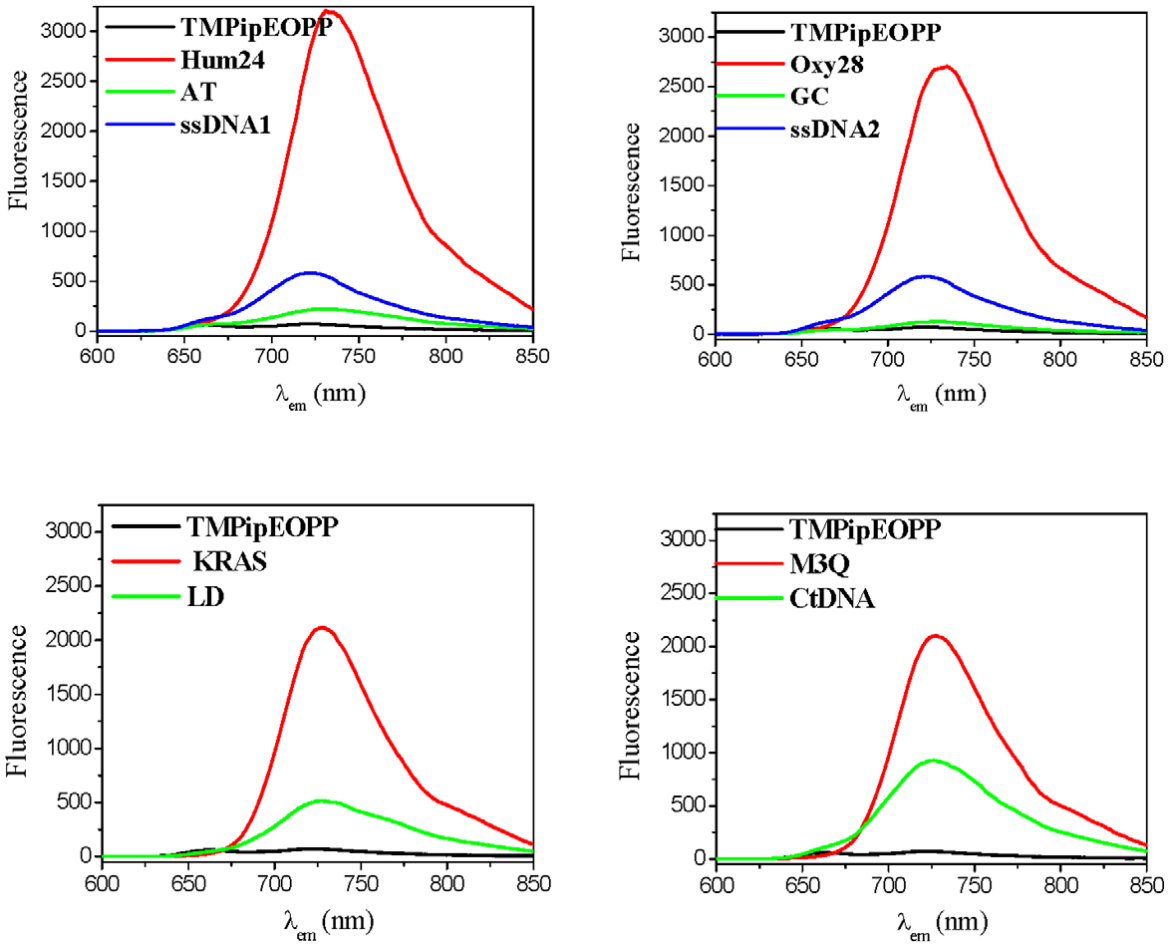
Fluorescence spectra of TMPipEOPP in the absence or presence of different DNAs when excited at 464 nm. [TMPipEOPP] = 5 µM. [DNA] = 10 µM (strand concentration). [CtDNA] = 240 µM (base concentration).

Using 464 or 700 nm as excitation wavelengths, the TMPipEOPP emission spectra in the absence or presence of different DNAs were compared. When excited at 464 nm, free TMPipEOPP was nearly non-fluorescent (Figure 6). However, the presence of G-quadruplexes greatly increased the fluorescence; for example, a 45-fold fluorescence increase was observed when 10 µM Hum24 was added. The weakest effect of the four G-quadruplexes was from 10 µM M3Q, but a 27-fold fluorescence increase was still observed. Although the presence of duplex or single-stranded DNA increased the fluorescence intensity of TMPipEOPP to some degree, the extent of fluorescence increase was much lower than from G-quadruplexes. For duplex and single-stranded DNAs, long duplex CtDNA gave the largest fluorescence increase, but this was only an 11-fold fluorescence increase. In the presence of 10 µM short duplex GC, nearly no fluorescence increase was observed compared to free TMPipEOPP. When excited at 700 nm, the different effects of different DNAs (G-quadruplexes versus duplex and single-stranded DNAs) were even greater (Figure S2).

In conclusion, compared with duplex and single-stranded DNAs, G-quadruplexes caused characteristic changes in the fluorescence signal of TMPipEOPP. (1) When excited at 422 nm, free TMPipEOPP and TMPipEOPP-DNA complexes displayed two emission peaks at 660 nm and 726 nm. However, free TMPipEOPP and the complexes formed with duplex or single-stranded DNAs had similar emission spectrum shapes, with the fluorescence intensity at the lower wavelength larger than the higher one. The complexes formed by G-quadruplex DNAs displayed a distinctively different spectrum shapes, with the fluorescence intensity at the higher wavelength is larger than the lower one. (2) With the emission wavelength at 726 nm, the strongest excitation peaks of free TMPipEOPP and its complexes with duplex or single-stranded DNAs were all around 422 nm, but complexes formed by G-quadruplex DNAs give three strong excitation peaks at 270, 464 and 700 nm, respectively. (3) When excited at 464 nm or 700 nm, free TMPipEOPP was nearly non-fluorescent, but G-quadruplexes increased the fluorescence to much higher levels, and G-quadruplexes had a much stronger ability to enhance the fluorescence of TMPipEOPP than duplex or single-stranded DNAs. These data indicate that perfect discrimination of G-quadruplexes from duplex and single-stranded DNAs can also be achieved according to the fluorescence spectrum changes of TMPipEOPP, and TMPipEOPP might be a good fluorescent probe for discriminating G-quadruplexes from duplex and single-stranded DNAs.

### Job Plot Analysis

**Figure 7:**
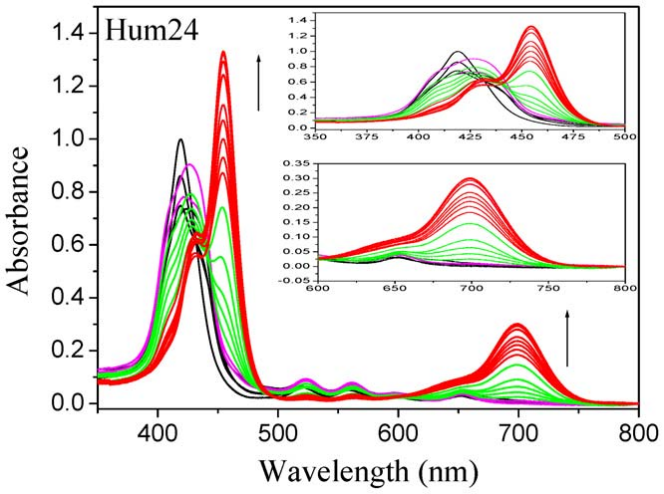
Absorption titration of TMPipEOPP with Hum24. [TMPipEOPP] = 5 µM. The Hum24 concentration increases from 0 to 50 µM.

To elucidate the binding interaction between DNAs and TMPipEOPP, absorbance titration experiments were conducted by collecting spectra of TMPipEOPP after addition of DNAs (Figure 7, Figure S3, S4, S5, and S6). Figure 7 shows a representative result of TMPipEOPP titration by Hum24. The absorbance spectrum changes of TMPipEOPP with Hum24 addition, can be divided into two steps. In the first step (black, magenta and green lines in Figure 7), Hum24 mainly affects the absorption peak at 419 nm. Hum24 addition resulted in a red shift from 419 to 430 nm and >35% hypochromicity of this peak (Table S2). However, the absorption signal at 455 and 700 nm was almost unchanged before the [Hum24]/[TMPipEOPP] ratio reached 0.5 (black and magenta lines in Figure 7). In the second step (green and red lines in Figure 7), Hum24 addition mainly caused changes in the absorption signals at 455 and 700 nm, and the absorption signal at 419 nm was almost not affected. With increasing Hum24 concentration, two new peaks appeared at 455 and 700 nm, and their absorption signals increased continuously. Similar results were obtained for TMPipEOPP titrations with M3Q, Oxy28 and KRAS (Figure S3, S4, and S5).

Based on these observations, we propose that two distinct binding modes occur between TMPipEOPP and G-quadruplexes at different [G-quadruplex]/[TMPipEOPP] ratios. To determine the number of molecules of bound TMPipEOPP binding per quadruplex, continuous variation analysis (Job plot) was performed (Figure S7, S8, S9, and S10). As for Hum24, M3Q and Oxy28, The results of Job plot analysis suggested a binding stoichiometry of two TMPipEOPP per G-quadruplex at low [G-quadruplex]/[TMPipEOPP] ratios, and a binding stoichiometry of two G-quadruplex per TMPipEOPP at high [G-quadruplex]/[TMPipEOPP] ratios. As for KRAS, the Job plot gave a binding stoichiometry of three TMPipEOPP per G-quadruplex at low [KRAS]/[TMPipEOPP] ratios (Figure S10). However, at high [KRAS]/[TMPipEOPP] ratios, the binding stoichiometry of TMPipEOPP to KRAS was also 1∶2. Similar results were obtained for Job plot analysis using fluorescence signals (Figure S11).

### Proposed binding modes between TMPipEOPP and G-quadruplexes

Generally, ligands interact with G-quadruplexes by three modes: end-stacking, intercalation, and outside binding [29]. Studies on the interaction of porphyrins with duplex DNAs suggest that typical intercalation causes a ≥15 nm red shift and a ≥35% hypochromicity of the porphyrin Soret band. External binding results in a ≤8 nm red shift and either hyperchromicity or ≤10% hypochromicity of the porphyrin soret band [30]. In our case, interactions between TMPipEOPP and G-quadruplexes displayed 11-nm red shifts and 36–52% hypochromicites at low [G-quadruplex]/[TMPipEOPP] ratios (Table S2). The hypochromicities were in the range for intercalative binding but the red shifts were not. Since reported red shifts (≥15 nm) and hypochromicities (≥35%) were for long pieces of duplex DNA [31], where end stacking is insignificant, one TMPipEOPP molecule might interact with G-quadruplexes through an end-stacking mode. Because the Scatchard analysis of the absorption titration data showed two TMPipEOPP molecules interact with one G-quadruplex at two types of sites, and the two binding interactions influence each other (Figure S12), the other TMPipEOPP molecule might interact with the G-quadruplexes in an outside-binding mode. For KRAS, two TMPipEOPP molecules might stack on the two ends of KRAS with one TMPipEOPP molecule binding to KRAS in an outside-binding mode, or one TMPipEOPP molecule stacking on one end of KRAS and two TMPipEOPP molecules binding to KRAS by outside binding.

At high [G-quadruplex]/[TMPipEOPP] ratios, a new absorption peak appeared around 455 nm. Compared to the previous Soret band of TMPipEOPP, a 36-nm red shift was observed. Combined with the binding stoichiometry of two G-quadruplexes per TMPipEOPP, a sandwich-like complex might be formed, with one TMPipEOPP molecule between the external planes of two quadruplexes. A similar complex structure was reported for TMPyP4. A binding stoichiometry of 1.5 molecules of TMPyP4 per quadruplex structure was observed by Wei et al. [32].

Based on our results, we propose a possible binding mechanism of TMPipEOPP to G-quadruplexes (Scheme S3). At low [G-quadruplex]/[TMPipEOPP] ratios, one G-quadruplex binds to two TMPipEOPP molecules. One TMPipEOPP molecule stacks at one end of the G-quadruplex and the other interacts with the quadruplex through an outside-binding mode. At high [G-quadruplex]/[TMPipEOPP] ratios, two G-quadruplexes bind to one TMPipEOPP molecule, with the TMPipEOPP molecule stacking between the ends of two G-quadruplexes in a sandwich-like mode.

## Discussion

Compared to well-studied prophyrins, for example TMPyP4 and TPrPyP4, TMPipEOPP shows a distinctively different change in UV-Vis absorption spectrum with the addition of G-quadruplexes. For TMPyP4 and TPrPyP4, continuous red shift and hypochromicity are observed with increasing G-quadruplex concentration. When the G-quadruplex concentration is sufficiently high, the absorption signal remains unchanged [21], [31], [33]. However, at high G-quadruplex concentrations, a further increase in G-quadruplex concentration causes the disappearance of the previous TMPipEOPP Soret band, and a new absorption band appears at a higher wavelength (∼455 nm), than the previous Soret band, and the red shift is as large as 36 nm. Simultaneously, a new absorption band appears at the NIR (Near Infrared Ray) region (∼700 nm). Increasing G-quadruplex concentration leads to continuous hyperchromicities of the two new bands. These results suggest that the binding interaction between TMPipEOPP and G-quadruplexes is more complicated than between TMPyP4 (or TPrPyP4) and G-quadruplexes. As a contrast, duplex and single-stranded DNAs could not cause similar absorption spectrum changes in TMPipEOPP (Figure S6), suggesting that the difference in the side arm substituents might give corresponding prophyrin derivatives different G-quadruplex selectivity for duplex and single-stranded DNAs.

G-quaruplexes also caused great changes in the fluorescence characteristics of TMPipEOPP. In the presence of G-quadruplexes, red shifts of 42 and 66 nm were observed for the excitation and emission wavelengths of TMPipEOPP. Another attractive feature was that a new excitation band (centered at 700 nm) emerged in the presence of G-quadruplexes. The excitation and emission peaks (centered at 726 nm) are all in the near-infrared region, which has less interaction with light rays in water and other tissue components, resulting in less light scattering and reduced background [33]. The unique fluorescence properties, combining with the high G-quadruplex selectivity over other DNA forms, making TMPipEOPP a potential luminescent G-quadruplex probes for *in vivo* detection of G-quadruplex DNA, for example G-quadruplex imaging and localization in living cells [8], [9].

In this work, the interactions between TMPipEOPP and four G-quadruplexes were investigated. These four G-quadruplexes each has only one G-quadruplex unit and belong to simple G-quadruplexes. At the end of human chromosome, there is a long (∼200 nt) single-stranded protrusion of TTAGGG repeats. It is reported that consecutive G-quadruplex structures, packed by several G-quadruplex units, would be formed [34]. The compounds that can selectively bind into the pocket between two adjacent G-quadruplex units, may be used as good telomeric G-quadruplex-stabilizing ligands and potent telomerase inhibitors. To date, only two classes of compounds have been reported as consecutive G-quadruplex structure-targeting agents [35], [36]. One is a chiral cyclic helicene, the other is a three side-chained triazatruxene derivative azatrux. In this paper, it has been suggested that TMPipEOPP molecule may stack between the ends of two G-quadruplexes in a sandwich-like mode. It is very possible that TMPipEOPP may be developed as the third agent targeting consecutive G-quadruplex structures. This finding informs our ongoing efforts to investigate the interactions between TMPipEOPP and consecutive G-quadruplexes.

In conclusion, we have investigated specific G-quadruplex recognition ability of porphyrin derivatives with large side arm substituents. To this end, a new cationic porphyrin derivative, TMPipEOPP, was synthesized and structurally characterized. Different from TMPyP4, a cationic porphyrin containing much smaller side arm substituents, TMPipEOPP can easily discriminate G-quadruplex from duplex and single-stranded DNA. Visual discrimination is also possible. G-quadruplexes can cause large changes in the UV-Vis absorption and fluorescence spectra of TMPipEOPP, but duplex and single-stranded DNA can not. A complicated binding interaction may occur between TMPipEOPP and G-quadruplexes. At a low [G-quadruplex]/[TMPipEOPP] ratio, one G-quadruplex binds two TMPipEOPP molecules by end-stacking and outside binding modes. At a high [G-quadruplex]/[TMPipEOPP] ratio, two G-quadruplexes bind to one TMPipEOPP molecule in a sandwich-like end-stacking mode. This finding suggests that TMPipEOPP may be developed as a consecutive G-quadruplex structure-targeting agent.

## Materials and Methods

### Materials and reagents

The oligonucleotides listed in Table 1 were purchased from Sangon Biotech. Co. Ltd. (Shanghai, China). The concentrations of the oligonucleotides were represented as single-stranded concentration unless otherwise noted. Single-stranded concentration was determined by measuring the absorbance at 260 nm. Molar extinction coefficient was determined using a nearest neighbour approximation (http://www.idtdna.com/analyzer/Applications/OligoAnalyzer), and the calculated molar extinction coefficients of these oligonucleotides were listed in Table 1. the concentration of CtDNA was represented as base concentration, which was determined by absorption spectroscopy using the molar absorption coefficient (6600 M^−1^•cm^−1^) at 260 nm. The solution gave a ratio of UV absorbance at 260 and 280 nm of ∼1.83∶1, indicating that the DNA was sufficiently free of protein. EDTA-2Na (Disodium ethylenediamine tetraacetic acid), Tris (tris(hydroxymethyl)aminomethane), KAc, N,N-Dimethylformamide (DMF) and CH_2_Cl_2_ were obtained from Sigma. 5,10,15,20-tetra-(4-hydroxyphenyl)porphyrin (THPP) was obtained from TCI Development Co., Ltd (Shanghai, China). 1-(2-Chloroethyl)piperidine hydrochloride was bought from Huai'an City East Chemical Factory (Jiangsu China). DMF was distilled over CaH_2_ before use. CH_2_Cl_2_ was distilled from CaH_2_ and stored over molecular sieves. Other chemical reagents were of analytical grade and used without further purification. Deionized and sterilized water (resistance >18 MΩ/cm) was used throughout the experiments.

### Physical methods

The NMR spectra were recorded on Bruker AV400 spectrometer operating for 1H NMR. Chemical shifts in the 1H NMR spectra are reported in ppm relative to the residual hydrogen atoms in the deuterated solvents: *δ* = 2.50 and 7.25 ppm for [D_6_]DMSO and CDCl_3_, respectively. Mass-spectroscopic analysis was performed on Bruker Autoflex III MALDI-TOF MS and LCQ Advantage MS detectors. Absorption spectra were measured on a TU-1901 UV-Vis spectrophotometer. Fluorescence spectra were measured on a Hitachimodel RF-4500 spectrofluorimeter. Circular dichroism (CD) spectra were measured on a Jasco J-715 spectropolarimeter.

### Synthesis of 5,10,15,20-tetra-{4-[2-(1-piperidinyl)ethoxy]phenyl} porphyrin (TPipEOPP) (1)

A suspension of THPP (108 mg, 0.16 mmol), 1-(2-chloroethyl)piperidine hydrochloride (236 mg, 1.28 mmol), and K_2_CO_3_ (310 mg, 2.24 mmol) in dry DMF (30 mL) was stirred for 72 h at room temperature under N_2_. Then the mixture was filtered. The red–brown precipitate was obtained and washed with DMF (5 mL) and diethyl ether (5 mL). The residue was dissolved in dichloromethane (100 mL) and washed with water. The organic layer was evaporated under reduced pressure. The resulting solid was isolated by chromatography on alumina (100–200 mesh) with ethyl acetate/methanol (v∶v = 50∶1). The first fraction was collected and the solvent was evaporated. Further purification was carried out by recrystallization from methanol/dichloromethane (v∶v = 1∶1). The purple-brown crystals of **1** were obtained in 52.8% yield (95 mg, 0.085 mmol). 1H NMR (400 MHz, CDCl_3_, 25°C, TMS): *δ* = −2.78 (s, 2H; pyrrole H), 1.59 (s, 8H; piperidine H), 1.81 (s, 16H; piperidine H), 2.80 (s, 16H; piperidine H), 3.09 (s, 8H; NCH_2_), 4.50(d, 8H; OCH_2_), 7.28 (d, 8H; Ph-H), 8.10 (d, 8H; Ph-H), 8.84 ppm (s, 8H; β-pyrrole H); ESI: m/z: calcd for C_72_H_81_N_8_O_4_+H^+^: 1123.47; found: 1123.66 [M+H^+^].

### Synthesis of 5,10,15,20-tetra-{4-[2-(1-methyl-1-piperidinyl)ethoxy]phenyl} porphyrin tetraiodide (TMPipEOPP•4I) (2)

To a suspension of **1** (58.4 mg, 0.052 mmol) in dry CH_2_Cl_2_ (30 mL) was added CH_3_I (15 mL, 0.24 mmol). The mixture was stirred under N_2_ and heated by using an oil bath at 40°C for 24 h. The solvent was evaporated and the resulting solid was washed with CH_2_Cl_2_ and diethyl ether in turn. **2** was obtained as a red-purple solid in 54% yield (48 mg, 0.028 mmol). 1H NMR (400 MHz, [D_6_]DMSO, 25°C, TMS): *δ* = −2.90 (s, 2H; pyrrole H), 1.66 (s, 8H; piperidine H), 1.96 (s, 16H; piperidine H), 3.31 (s, 12H; NCH_3_), 3.59 (m, 16H; piperidine H), 4.02 (s, 8H; NCH_2_), 4.79 (s, 8H; OCH_2_), 7.47 (d, 8H; Ph-H), 8.18 (d, 8H; Ph-H), 8.86 ppm (s, 8H; β-pyrrole H); ESI: m/z: calcd for [C_76_H_93_N_8_O_4_-4I]/4:295.9; found: 296.0 [M^+^-4I]/4.

### Crystal Structure Analysis of TPipEOPP•2.5MeOH

Crystallographic data were collected on a Bruker Smart CCD diffractometer at 113(2) K using graphite-monochromated Mok*α* radiation (λ = 0.71073 Å), and were corrected for Lorentz and polarization effects. The frames were integrated with the Bruker SAINT software package and the data were corrected for absorption using the program SASABS [37]. The structures were solved by direct methods using the program SHELXS-97 [38]. All non-hydrogen atoms were refined with anisotropic thermal parameters by full-matrix least-squares calculations on *F* ^2^ using the program SHELXL-97 [39]. Hydrogen atoms were inserted at calculated positions and constrained with isotropic thermal parameters. Crystal data: Monoclinic, *P*2(1)/*c*, *a* = 18.235(10) Å, *b* = 18.597(11) Å, *c* = 20.707(11) Å, *α* = 90°, *β* = 97.365(13)°, *γ* = 90°, *V* = 6964(7) Å^3^, *Z* = 4, *ρ* = 1.148 g•cm^−3^, *μ* = 0.074 mm^−1^, *F*(000) = 2588, crystal size 0.30×0.24×0.01 mm. A total of 52415 reflections collected (*θ* range of 1.48 to 25.02°), 12191 unique (*R*_int_ = 0.0750), *R*_1_ = 0.2060 (6443 reflections with *I*>*2σ(I)*) for 61 refined parameters and 818 restraints. Largest difference peak and hole 0.642 and −0.431 e•Å^−3^. The selected bond lengths and angles are listed in Table S1.

### Absorption spectroscopy

Absorption spectra were measured on a TU-1901 UV-Vis spectrophotometer with 1 cm-path-length micro quartz cell (40 µL, Starna Brand, England). Solutions containing 10 µM individual oligonucleotides (strand concentration) or 240 µM CtDNA (base concentration), 10 mM Tris-HCl buffer (pH = 7.0), 50 mM KCl and 1 mM EDTA-2Na were prepared. Each sample was heated to 95°C for 5 min to remove any aggregates, then cooled rapidly to 25°C and was allowed to incubate at 25°C for 30 min. After overnight incubation at 4°C, 5 µM of TMPipEOPP was added and the absorption spectra in the range of 350∼800 nm were recorded.

Absorption titration experiments were carried out by varying the DNA concentration and maintaining the TMPipEOPP concentration constant. Solutions containing 10 mM Tris-HCl buffer (pH = 7.0), 50 mM KCl, 1 mM EDTA-2Na and different DNA concentrations were prepared. Each sample was heated to 95°C for 5 min to remove any aggregates, then cooled rapidly to 25°C and was allowed to incubate at 25°C for 30 min. After overnight incubation at 4°C, 5 µM of TMPipEOPP was added and the absorption spectra in the range of 350∼800 nm were recorded.

The Job plot analysis was performed by systematic variation of the molar fraction of TMPipEOPP and DNA while keeping a constant total concentration of TMPipEOPP and DNA at 5 µM. The mixtures of TMPipEOPP and DNA were prepared as above. The absorption signals at 419, 430 and 700 nm were recorded.

### Fluorescence spectroscopy

Fluorescence spectra were measured on a Hitachimodel RF-4500 spectrofluorimeter with 1 cm-path-length micro quartz cell (40 µL, Starna Brand, England). Solutions containing 10 µM individual oligonucleotides (strand concentration) or 240 µM CtDNA (base concentration), 10 mM Tris-HCl buffer (pH = 7.0), 50 mM KCl and 1 mM EDTA-2Na were prepared. Each sample was heated to 95°C for 5 min to remove any aggregates, then cooled rapidly to 25°C and was allowed to incubate at 25°C for 30 min. After overnight incubation at 4°C, 5 µM of TMPipEOPP was added. Fixing the excitation or emission wavelengths, corresponding emission or excitation spectra were collected at room temperature: (1) The excitation wavelength was fixed at 422 nm and the emission spectra were collected in the range of 600∼850 nm; (2) The emission wavelength was fixed at 726 nm and the excitation spectra were collected in the range of 200∼720 nm; (3) The excitation wavelength was fixed at 464 nm and the emission spectra were collected in the range of 600∼850 nm; (4) The excitation wavelength was fixed at 700 nm and the emission spectra were collected in the range of 710∼900 nm.

The Job plot analysis was performed by systematic variation of the molar fraction of TMPipEOPP and DNA while keeping a constant total concentration of TMPipEOPP and DNA at 5 µM. The mixtures of TMPipEOPP and DNA were prepared as above. Setting the excitation wavelength at 700 nm, the fluorescence signal at 726 nm was recorded.

### Circular dichroism (CD) spectroscopy

3 mL reaction mixture was prepared in 10 mM Tris-HCl buffer (pH = 7.0) containing 10 mM KCl and 1 µM individual DNA oligonculeotide. The mixture was heated at 95°C for 5 min, cooled slowly to 25°C and then incubated at 4°C overnight. CD spectrum of the mixture was recorded between 200 and 320 nm in 1 mm path length cuvettes on a Jasco J-715 spectropolarimeter. Spectra were averaged from 3 scans, which were recorded at 100 nm/min with a response time of 1 s and a bandwith of 1.0 nm.

## Supporting Information

Figure S1 **CD spectra of M3Q, Oxy28, KRAS and Hum24.**
(TIF)

Figure S2 **Fluorescence spectra of TMPipEOPP in the absence or presence of different DNAs when excited at 700 nm.** [TMPipEOPP] = 5 µM. [DNA] = 10 µM (strand concentration). [CtDNA] = 240 µM (base concentration).
(TIF)

Figure S3 **Absorption titration of TMPipEOPP with M3Q.** [TMPipEOPP] = 5 µM. The M3Q concentration increases from 0 to 20 µM.
(TIF)

Figure S4 **Absorption titration of TMPipEOPP with Oxy28.** [TMPipEOPP] = 5 µM. The Oxy28 concentration increases from 0 to 50 µM.
(TIF)

Figure S5 **Absorption titration of TMPipEOPP with KRAS.** [TMPipEOPP] = 5 µM. The KRAS concentration increases from 0 to 50 µM.
(TIF)

Figure S6 **Absorption titration of TMPipEOPP with AT, GC, LD, CtDNA, ssDNA1 or ssDNA2.** [TMPipEOPP] = 5 µM. The concentration of each DNA is shown in the figure. The concentrations of AT, GC, LD, ssDNA1 and ssDNA2 are represented as single-stranded concentrations. The concentration of CtDNA is represented as base concentration.
(TIF)

Figure S7 **Job plots resulting from the method of continuous variation analysis for TMPipEOPP with Hum24.** [TMPipEOPP]+[Hum24] = 5 µM.
(TIF)

Figure S8 **Job plots resulting from the method of continuous variation analysis for TMPipEOPP with M3Q.** [TMPipEOPP]+[M3Q] = 5 µM.
(TIF)

Figure S9 **Job plots resulting from the method of continuous variation analysis for TMPipEOPP with Oxy28.** [TMPipEOPP]+[Oxy28] = 5 µM.
(TIF)

Figure S10 **Job plots resulting from the method of continuous variation analysis for TMPipEOPP with KRAS.** [TMPipEOPP]+[KRAS] = 5 µM.
(TIF)

Figure S11 **Job plot analysis using the fluorescence signals at 726 nm.** [TMPipEOPP]+[G-quadruplex] = 5 µM. λ_ex_ = 700 nm.
(TIF)

Figure S12 **Scatchard plots for TMPipEOPP with individual G-quadruplexes.** *r* is moles of bound TMPipEOPP per mole of G-quadruplex.
(TIF)

Scheme S1 **The chemical structures of TMPyP4 and TMPipEOPP.**
(TIF)

Scheme S2 **The synthetic route of TMPipEOPP.**
(TIF)

Scheme S3 **The proposed binding modes between TMPipEOPP and G-quadruplex at different [G-quadruplex]/[TMPipEOPP] ratios.**
(TIF)

Table S1 **Selected bond lengths(Å) and angles(°) of TPipEOPP•2.5MeOH.**
(DOC)

Table S2 **Soret band hypochromicity caused by the titrations of TMPipEOPP with oligonucleotides.**
(DOC)

## References

[1] Huppert JL (2008). Four-stranded nucleic acids: structure, function and targeting of G-quadruplexes.. Chem Soc Rev.

[2] Patel DJ, Phan AT, Kuryavyi V (2007). Human telomere, oncogenic promoter and 5′-UTR G-quadruplexes: diverse higher order DNA and RNA targets for cancer therapeutics.. Nucleic Acids Res.

[3] Balasubramanian S, Hurley LH, Neidle S (2011). Targeting G-quadruplexes in gene promoters: a novel anticancer strategy?. Nat Rev Drug Discov.

[4] Tran PLT, Mergny J-L, Alberti P (2010). Stability of telomeric G-quadruplexes.. Nucleic Acids Res.

[5] Murat P, Singh Y, Defranoq E (2011). Methods for investigating G-quadruplex DNA/ligand interactions.. Chem Soc Rev.

[6] Balasubramanian S, Neidle S (2009). G-quadruplex nucleic acids as therapeutic targets.. Curr Opin Chem Biol.

[7] Lane AN, Chaires JB, Gray RD, Trent JO (2008). Stability and kinetics of G-quadruplex structures.. Nucleic Acids Res.

[8] Lu Y-J, Yan S-C, Chan F-Y, Zou L, Chung W-H (2011). Benzothiazole-substituted benzofuroquinolinium dye: a selective switch-on fluorescent probe for G-quadurplex.. Chem Commun.

[9] Alzeer J, Vummidi BR, Roth PJC, Luedtke NW (2009). Guanidinium-modified phthalocyanines as high-affinity G-quadruplex fluorescent probes and transcriptional regulators.. Angew Chem Int Ed.

[10] Romera C, Bombarde O, Bonnet R, Gomez D, Dumy P (2011). Improvement of porphyrins for G-quadruplex DNA targeting.. Biochimie.

[11] Bhasikuttan AC, Mohanty J, Pal H (2007). Interaction of malachite green with guanine-rich single-stranded DNA: preferential binding to a G-quadruplex.. Angew Chem Int Ed.

[12] Kong D-M, Ma Y-E, Wu J, Shen H-X (2009). Discrimination of G-quadruplexes from duplex and single-stranded DNAs with fluorescence and energy-transfer fluorescence spectra of crystal violet.. Chem Eur J.

[13] Teulade-Fichou M-P, Carrasco C, Guittat L, Bailly C, Alberti P (2003). Selective recognition of G-quadruplex telomeric DNA by a bis(quinacridine) macrocycle.. J Am Chem Soc.

[14] Yang P, De Cian A, Teulade-Fichou M-P, Mergny J-L, Monchaud D (2009). Engineering bisquinolinium/thiazole orange conjugates for fluorescent sensing of G-quadruplex DNA.. Angew Chem Int Ed.

[15] Arthanari H, Basu S, Kawano TL, Bolton PH (1998). Fluorescent dyes specific for quadruplex DNA.. Nucleic Acids Res.

[16] Guo J-H, Zhu L-N, Kong D-M, Shen H-X (2009). Triphenylmethane dyes as fluorescent probes for G-quadruplex recognition.. Talanta.

[17] Lombardo CM, Martínez IS, Haider S, Gabelica V, De Pauw E (2010). Structure-based design of selective high-affinity telomeric quadruplex-binding ligands.. Chem Commun.

[18] Nielsen MC, Ulven T (2010). Macrocyclic G-quadruplex ligands.. Curr Med Chem.

[19] Yamashita T, Uno T, Ishikawa Y (2005). Stabilization of guanine quadruplex DNA by the binding of porphyrins with cationic side arms.. Bioorg Med Chem.

[20] Wheelhousr RT, Sun D, Han H, Han FX, Hurley LH (1998). Cationic porphyrins as telomerase inhibitors: the interaction of Tetr-(N-methyl-4-pyridyl)porphine with G-quadruplex DNA.. J Am Chem Soc.

[21] Martino L, Pagano B, Fotticchia I, Neidle S, Giancola C (2009). Shedding light on the interaction between TMPyP4 and human telomeric quadruplexes.. J Phys Chem B.

[22] Wei C, Wang L, Jia G, Zhou J, Han G (2009). The binding mode of porphyrins with cation side arms to (TG_4_T)4 G-quadruplex: spectroscopic evidence.. Biophys Chem.

[23] Wei C, Jia G, Yuan J, Feng Z, Li C (2006). A spectroscopic study on the interactions of porphyrin with G-quadruplex DNAs.. Biochemistry.

[24] Ren J, Chaires JB (1999). Sequence and structural selectivity of nucleic acid binding ligands.. Biochemistry.

[25] Dixon IM, Lopez F, Esteve JP, Tejera AM, Blasco MA (2005). Porphyrin derivatives for telomere binding and telomerase inhibition.. Chembiochem.

[26] Dixon IM, Lopez F, Tejera AM, Estève J-P, Blasco MA (2007). A G-quadruplex ligand with 1000-fold selectivity over duplex DNA.. J Am Chem Soc.

[27] Bennett M, Krah A, Wien F, Garman E, McKenna R (2000). A DNA-porphyrin minor-groove complex at atomic resolution: the structural consequence of porphyrin ruffling.. Proc Natl Acad Sci U S A.

[28] Vialas C, Pratviel G, Meunier B (2000). Oxidative damage generated by an oxo-metalloporphyrin onto the human telomeric sequence.. Biochemistry.

[29] Pan J, Zhang S (2009). Interaction between cationic zinc porphyrin and lead ion induced telomeric guanine quadruplexes: evidence for end-stacking.. J Biol Inorg Chem.

[30] Pasternack RF, Gibbs EJ, Villafranca JJ (1983). Interactions of porphyrins with nucleci acids.. Biochemsitry.

[31] Wei C, Wang J, Zhang M (2010). Sepctroscopic study on the binding of porphyrins to (G_4_T_4_G_4_)4 parallel G-quadruplex.. Biophys Chem.

[32] Wei C, Jia G, Zhou J, Han G, Li C (2009). Evidence for the binding mode of porphyrins to G-quadruplex DNA.. Phys Chem Chem Phys.

[33] Leopoldo M, Lacivita E, Berardi F, Perrone R (2009). Developments in fluorescent probes for receptor research.. Drug Discov Today.

[34] Yu H-Q, Miyoshi D, Sugimoto N (2006). Characterization of structure and stability of long telomeric DNA G-quadruplexes.. J Am Chem Soc.

[35] Shinohara K-i, Sannohe Y, Kaieda S, Tanaka K-i, Osuga H (2010). A chiral wedge molecule inhibits telomerase activity.. J Am Chem Soc.

[36] Cummaro A, Fottichia I, Franceschin M, Giancola C, Petraccone L (2011). Binding properties of human telomeric quadruplex multimers: a new route for drug design.. Biochimie.

[37] Madison WI, SMART (control) and SAINT (integration) software, Bruker Analytical X-ray Systems (1994).

[38] Sheldrick GM (1997).

[39] Sheldrick GM (1997).

