# Supplementary figures and images for: A New Cationic Porphyrin Derivative (TMPipEOPP) with Large Side Arm Substituents: A Highly Selective G-Quadruplex Optical Probe
Source: PLoS One. 2012 May 22;7(5):e35586. doi: 10.1371/journal.pone.0035586 (PMC3358308; doi:10.1371/journal.pone.0035586)

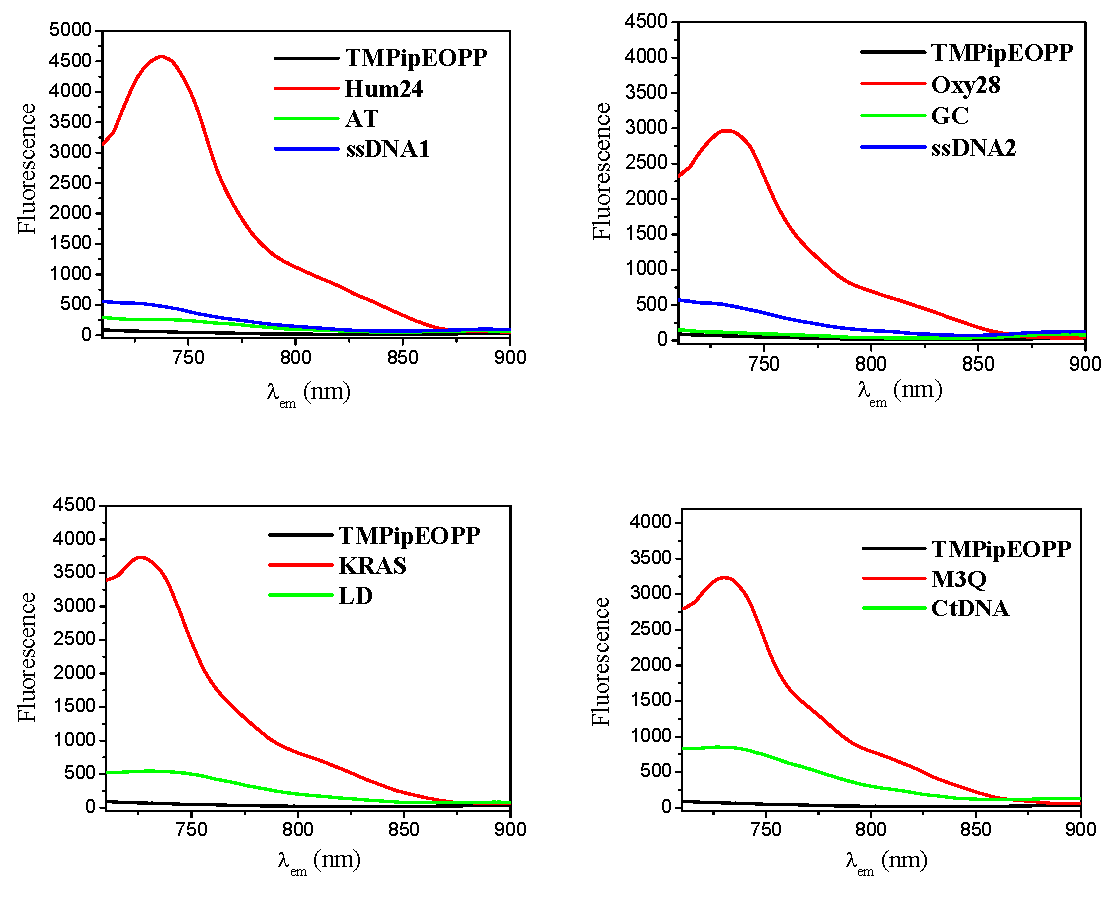

Supplement: Figure S2 — Fluorescence spectra of TMPipEOPP in the absence or presence of different DNAs when excited at 700 nm. [TMPipEOPP] = 5 µM. [DNA] = 10 µM (strand concentration). [CtDNA] = 240 µM (base concentration). (TIF) [file pone.0035586.s002.tif]

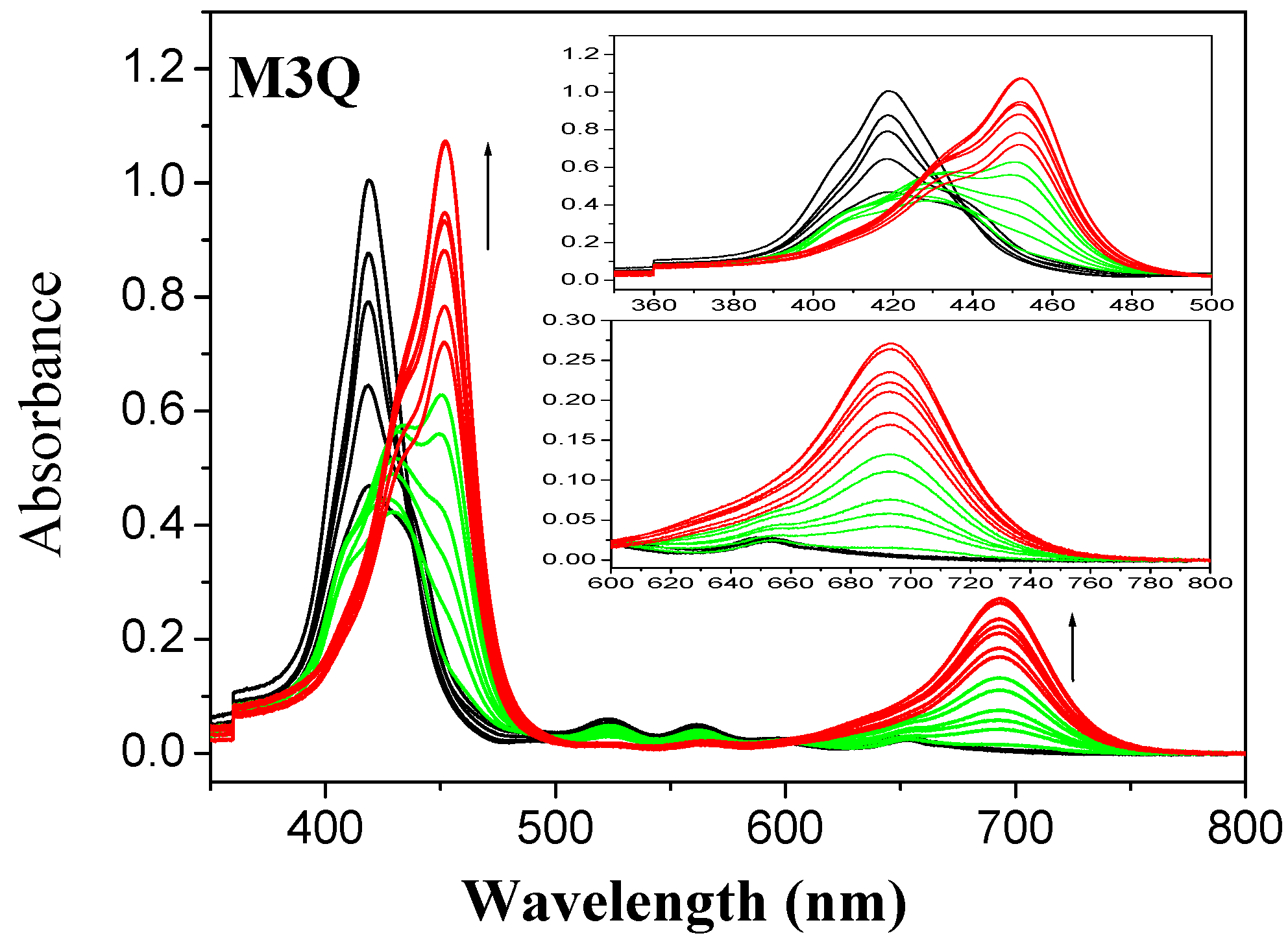

Supplement: Figure S3 — Absorption titration of TMPipEOPP with M3Q. [TMPipEOPP] = 5 µM. The M3Q concentration increases from 0 to 20 µM. (TIF) [file pone.0035586.s003.tif]

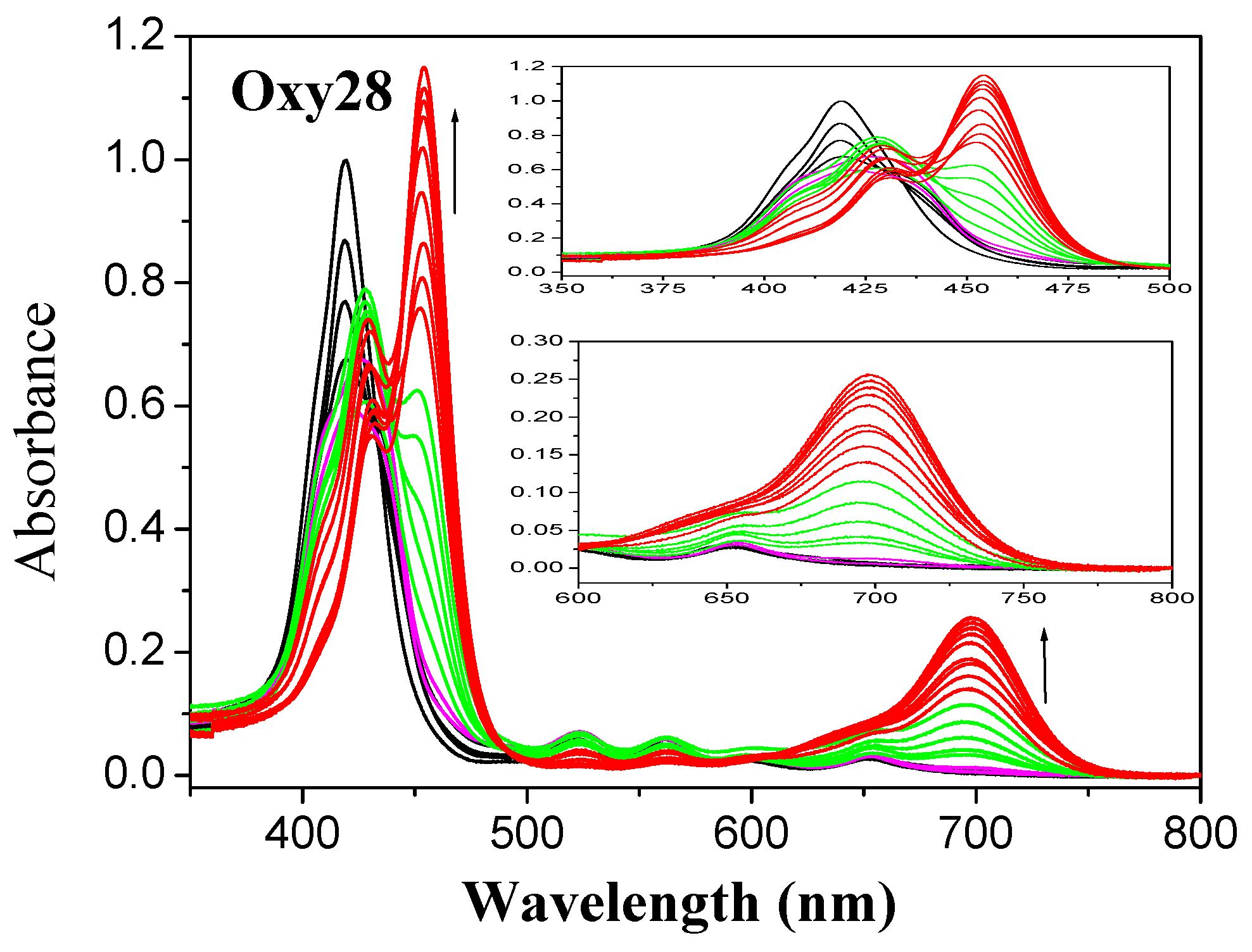

Supplement: Figure S4 — Absorption titration of TMPipEOPP with Oxy28. [TMPipEOPP] = 5 µM. The Oxy28 concentration increases from 0 to 50 µM. (TIF) [file pone.0035586.s004.tif]

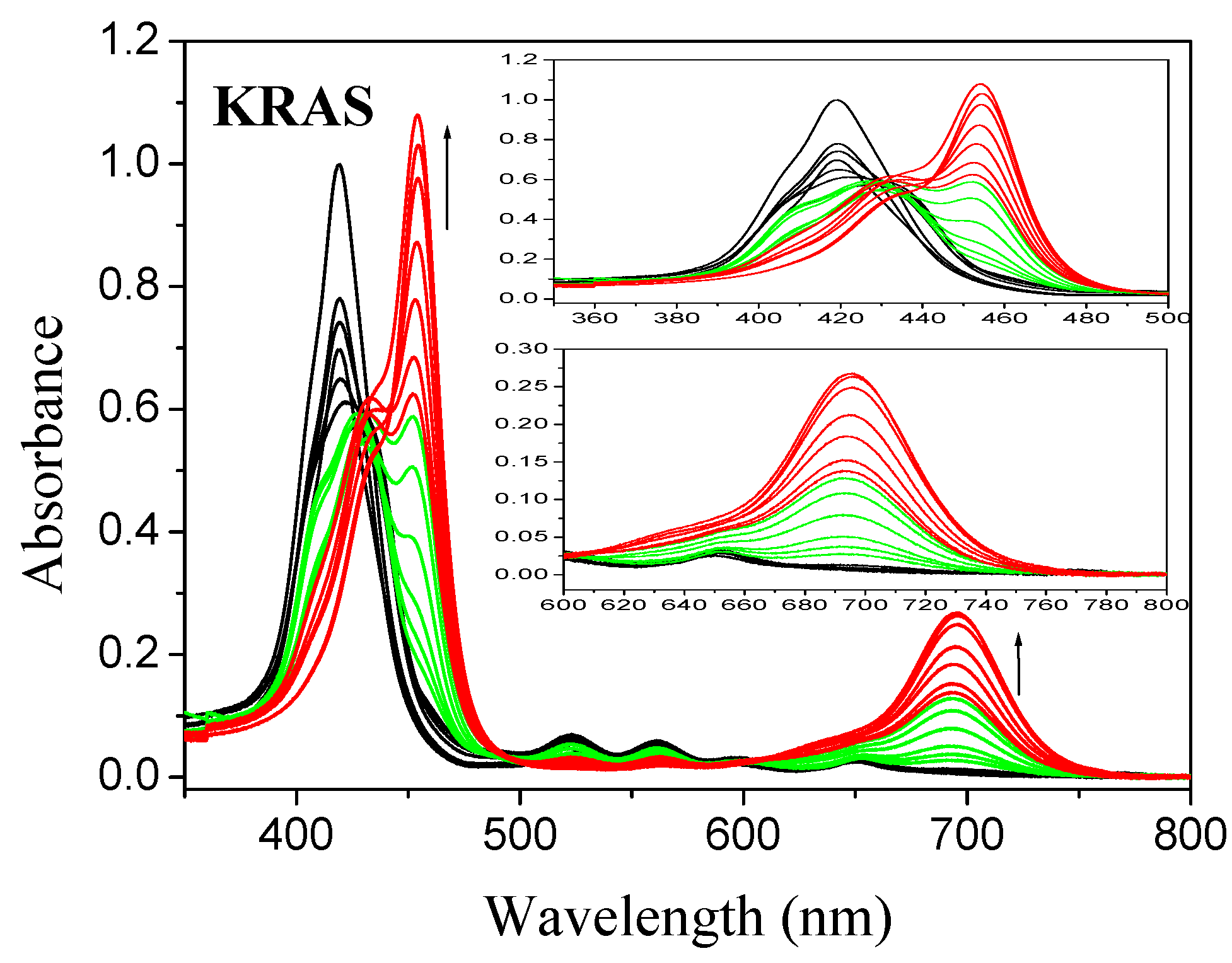

Supplement: Figure S5 — Absorption titration of TMPipEOPP with KRAS. [TMPipEOPP] = 5 µM. The KRAS concentration increases from 0 to 50 µM. (TIF) [file pone.0035586.s005.tif]

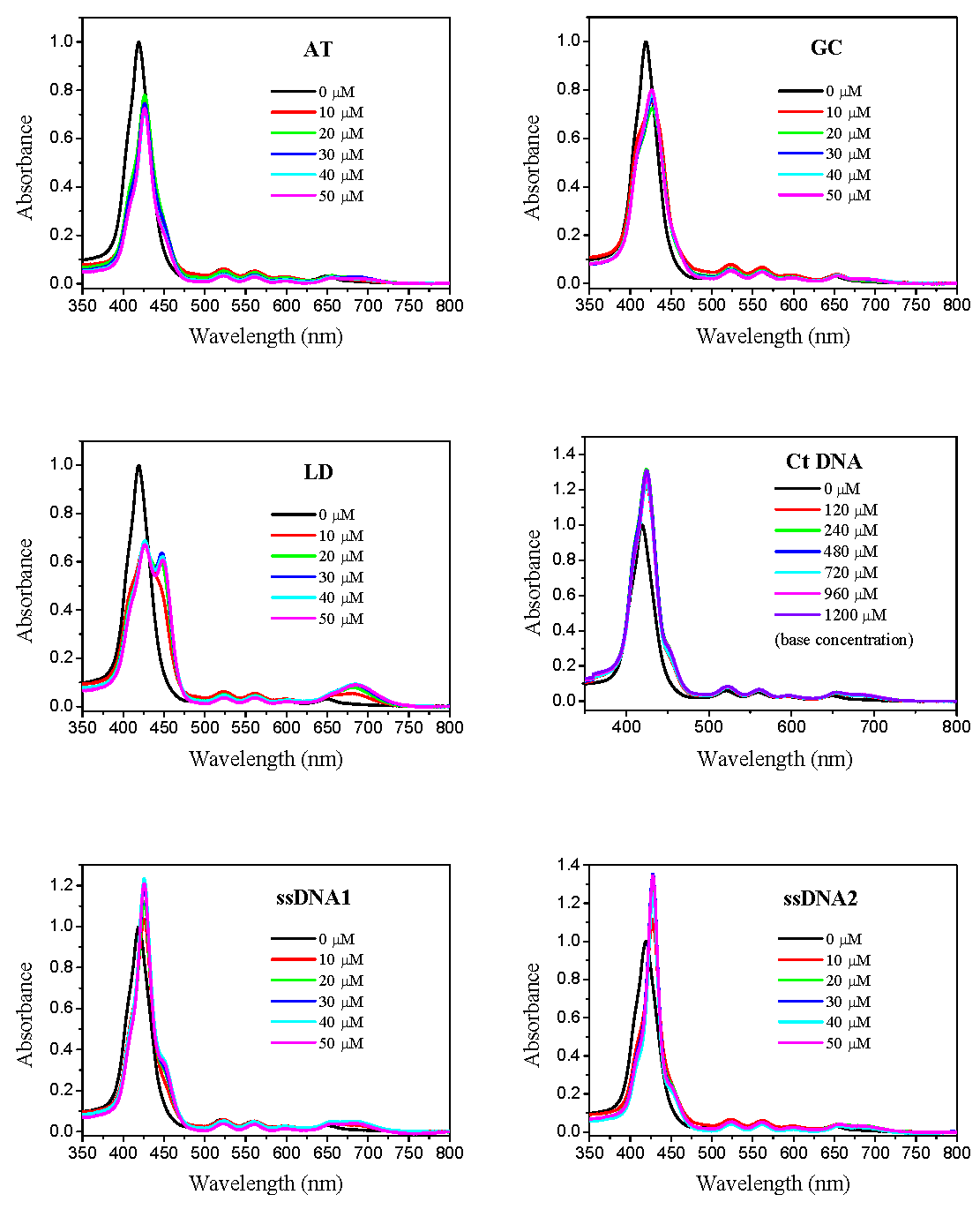

Supplement: Figure S6 — Absorption titration of TMPipEOPP with AT, GC, LD, CtDNA, ssDNA1 or ssDNA2. [TMPipEOPP] = 5 µM. The concentration of each DNA is shown in the figure. The concentrations of AT, GC, LD, ssDNA1 and ssDNA2 are represented as single-stranded concentrations. The concentration of CtDNA is represented as base concentration. (TIF) [file pone.0035586.s006.tif]

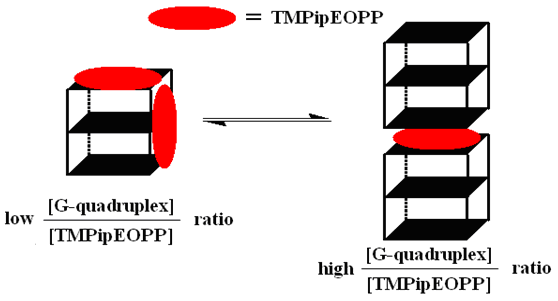

Supplement: Scheme S3 — The proposed binding modes between TMPipEOPP and G-quadruplex at different [G-quadruplex]/[TMPipEOPP] ratios. (TIF) [file pone.0035586.s015.tif]
