# Supplementary material for: A New Cationic Porphyrin Derivative (TMPipEOPP) with Large Side Arm Substituents: A Highly Selective G-Quadruplex Optical Probe
Source: PLoS One. 2012 May 22;7(5):e35586. doi: 10.1371/journal.pone.0035586 (PMC3358308; doi:10.1371/journal.pone.0035586)
Supplement: Table S1 — Selected bond lengths(Å) and angles(°) of TPipEOPP•2.5MeOH. (DOC) [file pone.0035586.s016.doc]

**Table S1**. **Selected bond lengths(Å) and angles(°) of TPipEOPP∙2.5MeOH.**

| Porphyrin core |  |  |  |
| --- | --- | --- | --- |
| N-C_α_ |  |  |  |
| N(1)-C(4) | 1.382(9) | N(2)-C(16) | 1.386(9) |
| N(1)-C(1) | 1.395(8) | N(2)-C(19) | 1.413(9) |
| N(3)-C(11) | 1.379(8) | N(4)-C(9) | 1.347(9) |
| N(3)-C(14) | 1.387(9) | N(4)-C(6) | 1.377(10) |
| N-H |  |  |  |
| N(1)-H(1) | 0.8996 | N(3)-H(3) | 0.8800 |
| C_α_-C_β_ |  |  |  |
| C(1)-C(2) | 1.408(11) | C(3)-C(4) | 1.444(11) |
| C(6)-C(7) | 1.489(11) | C(8)-C(9) | 1.431(10) |
| C(11)-C(12) | 1.423(10) | C(13)-C(14) | 1.424(10) |
| C(16)-C(17) | 1.499(11) | C(18)-C(19) | 1.387(10) |
| C_β_-C_β_ |  |  |  |
| C(2)-C(3) | 1.385(10) | C(7)-C(8) | 1.355(11) |
| C(12)-C(13) | 1.349(9) | C(17)-C(18) | 1.293(10) |
| C_α_-N-C_α_ |  |  |  |
| C(4)-N(1)-C(1) | 111.9(6) | C(16)-N(2)-C(19) | 106.5(7) |
| C(11)-N(3)-C(14) | 109.9(7) | C(9)-N(4)-C(6) | 105.2(6) |
| N-C_α_-C_β_ |  |  |  |
| N(1)-C(1)-C(2) | 105.5(7) | N(1)-C(4)-C(3) | 105.1(7) |
| N(2)-C(16)-C(17) | 104.4(8) | N(2)-C(19)- C(18) | 110.4(8) |
| N(3)-C(11)-C(12) | 105.5(7) | N(3)-C(14)-C(13) | 107.0(6) |
| N(4)-C(6)-C(7) | 109.9(8) | N(4)-C(9)-C(8) | 112.5(8) |
| Side arm |  |  |  |
| C(ph)-O |  |  |  |
| O(1)-C(24) | 1.365(8) | O(3)-C(50) | 1.359(8) |
| O(2)-C(37) | 1.369(12) | O(4)-C(63) | 1.387(11) |
| C(alk)-O |  |  |  |
| O(1)-C(27) | 1.464(9) | O(3)-C(53) | 1.456(9) |
| O(2)-C(40) | 1.342(13) | O(4)-C(66) | 1.470(10) |
| O(ph)-C-C-N(pip) |  |  |  |
| O(1)-C(27)-C(28)-N(5) | 88.8(9) | O(3)-C(53)-C(54)-N(7) | 78.7(10) |
| O(2)-C(40)-C(41)-N(6) | 62.0(14) | O(4)-C(66)-C(67)-N(8) | 66.9(10) |
