# Supplementary material for: A New Cationic Porphyrin Derivative (TMPipEOPP) with Large Side Arm Substituents: A Highly Selective G-Quadruplex Optical Probe
Source: PLoS One. 2012 May 22;7(5):e35586. doi: 10.1371/journal.pone.0035586 (PMC3358308; doi:10.1371/journal.pone.0035586)
Supplement: Table S2 — Soret band hypochromicity caused by the titrations of TMPipEOPP with oligonucleotides. (DOC) [file pone.0035586.s017.doc]

**Table S2. Soret band hypochromicity caused by the titrations of TMPipEOPP** with oligonucleotides

| oligonucleotides | % hypochromicity |
| --- | --- |
| Hum24 | 36.76 ± 0.71 |
| KRAS | 52.64 ± 0.66 |
| Oxy28 | 41.27 ± 0.05 |
| M3Q | 41.95 ± 2.17 |
| AT | 25.80 ± 2.57 |
| GC | 23.92 ± 2.92 |
| LD | 32.93 ± 0.73 |

The % hypochromicity of the Soret band was determined using the following equation:

$\%hypochromicity=[(\varepsilon_{free}-\varepsilon_{bound})/\varepsilon_{free}]\times100$ (Eq1)

Where $\varepsilon_{free}$ and $\varepsilon_{bound}$ are the extinction coefficients for the free TMPipEOPP and bound TMPipEOPP, respectively. The calculation of $\varepsilon_{free}$ and $\varepsilon_{bound}$can refer to the reference [1].

**References**

1. Keating LR, Szalai VA (2004) Parallel-stranded guanine quadruplex interactions with a copper cationic porphyrin. Biochemistry 43: 15891-15900.
